# The organizational impact of value-based health care: structure-related opportunities and challenges of condition-based units integrated within functional hospital structures

**DOI:** 10.1108/JHOM-10-2025-0699

**Published:** 2026-06-24

**Authors:** Femke Meijer, Paul Van der Nat, Dirk Vriens, Patrick Vermeulen, Dorine van Staalduinen

**Affiliations:** Department of Value-Based Healthcare, St Antonius Hospital, Nieuwegein, The Netherlands; Department of IQ Health, Radboud University Medical Center, Nijmegen, The Netherlands; Martini Hospital, Groningen, The Netherlands; Institute for Management Research, Radboud University Nijmegen, Nijmegen, The Netherlands; Santeon, Utrecht, The Netherlands

**Keywords:** Condition-based units, Condition-based organizing, Organization structure, Structure dimensions, Value-based health care

## Abstract

**Purpose:**

Value-based health care proposes that hospitals implement condition-based units (CBUs) to enhance patient value and foster collaboration among professionals. Although CBUs are proposed as an alternative to traditional functional hospital structures, many hospitals insert them within their existing functional structures. Using structural design theory as a framework, this study aims to examine the structure-related opportunities and challenges associated with CBUs integrated within functional hospital structures in order to understand how this integration affects their ability to improve patient value and collaboration.

**Design/methodology/approach:**

A qualitative multiple case study was conducted with members of 6 CBUs across 2 Dutch hospitals, involving 26 interviews analyzed thematically.

**Findings:**

Our findings show that the functional structure provides CBUs with resources and specialized expertise, including support on project management and data analysis. However, integrating CBUs within the functional structure can lead to ambiguity in decision-making authority within CBU processes, involve multiple stakeholders in CBU-related decisions and generate tensions between departmental and CBU goals.

**Practical implications:**

We recommend that hospital managers adopt a learning-oriented, experimental approach to gradually integrate care delivery and decision-making within CBUs, while positioning the functional structure as a supportive infrastructure.

**Originality/value:**

By identifying structure-related opportunities and challenges, this study addresses the lack of understanding regarding the consequences of implementing CBUs within functional hospital structures and indicates the need for hospitals to take additional measures to leverage opportunities and address associated challenges to maximize the potential of CBUs.

## Introduction

An increasing number of hospitals are reorganizing their healthcare delivery, guided by the principles of value-based health care (VBHC) (Daniels *et al*., 2022; Fernández-Salido *et al*., 2024; Steinmann *et al*., 2022). The fragmented nature of care processes and complex management in the traditional hospital structure often lead to problems such as delays, duplication of care, medication errors and, ultimately, poor patient outcomes (Christensen *et al*., 2009; Fiorio *et al*., 2018; Porter and Lee, 2021). As an alternative to the traditional functional hospital structure, Porter and Lee (2013, 2021) advocate a shift toward a condition-based organizational structure, where both clinical and non-clinical staff within condition-based units (CBUs) are collectively responsible for the entire care cycle of a medical condition. This approach requires professionals to collaborate in delivering care for specific conditions, such as prostate cancer, while simultaneously improving patient value, defined as patient outcomes relative to costs (Porter and Lee, 2013, 2021; Steinmann *et al*., 2022). Earlier studies indicate that a CBU enhances interdisciplinary collaboration and coordination among healthcare professionals, reduces waste and duplication in care and facilitates insight into outcomes and costs (Borde *et al*., 2023; Fernández-Salido *et al*., 2024; Kynoch *et al*., 2025; Van Staalduinen *et al*., 2023).

In practice, condition-based organizations take different forms. Wiersema *et al*. (2023) identified four archetypes of CBUs, namely, (*1) multidisciplinary project teams, (2) matrix organizations, (3) integrated practice units (IPUs) and (4) independent treatment centers (ITCs).* In the first two archetypes, the functional structure remains dominant, even though this seems at odds with the notion that CBUs are thought of as an alternative to these structures. In these cases, CBUs are responsible for improving the care process for a specific condition in *multidisciplinary project teams* and for both outcomes and costs of the care process in a *matrix organization*. Both forms operate in close alignment with the hospital's broader functional structure. In contrast, in archetypes three and four, the IPU or ITC assumes full responsibility for care delivery, thereby effectively replacing the traditional functional structure. These last two archetypes align more closely with the condition-based approach described by Porter and Lee (2021).

Current practice shows that hospitals mostly adhere to archetype one or two, integrating CBUs *within* the traditional functional hospital structure. In practice, fully separating a CBU from the broader hospital organization seems unfeasible due to practical and institutional constraints (Daniels *et al*., 2022; Steinmann *et al*., 2022; Wiersema *et al*., 2023). For example, specialized and capital-intensive services, such as radiology, are required across multiple conditions, making it financially and logistically infeasible to embed them within each CBU to achieve complete independence (Keswani *et al*., 2016). In addition, the existing reimbursement infrastructure may pose financial challenges for the establishment of fully independent CBUs.

While prior research has described CBUs in hospitals (e.g. Daniels *et al*., 2022; Steinmann *et al*., 2022; Wiersema *et al*., 2023), less is known about their actual functioning within functional hospital structures. It remains unclear how hospitals can integrate CBUs within the functional hospital structure so that they can optimize patient value and enhance collaboration among professionals (Wiersema *et al*., 2023). As a result, hospitals lack an understanding of the consequences of implementing CBUs within the current functional structure and may be unable to decide whether this is a suitable approach or to take appropriate measures to circumvent (some of) the associated challenges.

To address this gap, the present study aims to examine the following research question: *What structure-related opportunities and challenges arise from the integration of CBUs within the functional structure of hospitals?* To answer this question, we draw on structural design theory (hereafter referred to as design theory). It enables us to examine the tasks and responsibilities of CBUs integrated within the functional structure and to understand how and why this integration may create opportunities or challenges.

## Theory

### Introduction to design theory

Design theory examines how organizational structures influence performance outcomes (Burton and Obel, 2018; Joseph and Sengul, 2024). An organizational structure can be understood as the allocation and coordination of tasks and responsibilities across individuals, teams and departments (Mintzberg, 1979). There are different types of organizational structures, which can be described and characterized by structure dimensions, such as specialization and centralization (Mintzberg, 1979; Vriens *et al*., 2018). In literature on organizational structures, authors such as De Sitter (1994) and Mintzberg (1979) distinguish different structure dimensions. In this study, we focus on three – functional concentration, specialization and centralization – which we consider relevant for the analysis of CBUs within functional hospital structures. In our view, these dimensions capture the core structural aspects of CBUs as described by Porter and Lee (2021) and enable the characterization of structure-related opportunities and challenges expected and experienced by CBUs integrated within functional structures.

*Functional concentration* refers to the extent to which the operational tasks of individuals or teams are dedicated to a specific order type (De Sitter, 1994; Vriens *et al*., 2018). An order type may refer to a certain product or service, a specific geographical area, or a particular type of customer. In the context of healthcare, a high degree of functional concentration indicates that tasks are associated with many, and potentially all, medical conditions or patient groups (Christensen *et al*., 2009). A low level of functional concentration indicates that tasks are linked to a few medical conditions or patient groups or even a single one. For instance, tasks in an outpatient clinic may cover multiple conditions, such as breast, prostate and colorectal cancers (e.g. relatively high functional concentration), or focus exclusively on a single condition such as an outpatient clinic dedicated to breast cancer (e.g. low value). Porter and Lee (2021) suggest that in the context of a condition-based organization, CBUs are ideally organized around well-defined medical conditions or patient groups. This reflects a need for low functional concentration at the level of CBUs.

*Specialization* concerns the degree to which tasks within a process are divided into sub-tasks (De Sitter, 1994; Mintzberg, 1979). Tasks can be subdivided into sub-tasks that are part of the primary process (De Sitter, 1994; Mintzberg, 1979), or into sub-tasks addressing specific aspects of a task, such as preparatory activities (e.g. scheduling or planning), support activities (e.g. administrative activities) or production activities (e.g. clinical work) (De Sitter, 1994). A high degree of specialization indicates that tasks cover a small part of the primary process or a specific aspect of it, whereas a low degree of specialization indicates that tasks encompass multiple steps of the primary process and cover all aspects (e.g. including preparatory and supportive activities). For instance, a department may be solely responsible for providing postoperative care or information technology (IT) support (e.g. high specialization), or it may encompass a broader range of responsibilities, such as diagnosing, preparing for surgery, providing postoperative care and performing supportive activities, such as internal logistics or technical services (e.g. low specialization). The literature on VBHC emphasizes that CBUs are preferably responsible for providing care and implementing improvements throughout the entire care cycle (Porter and Lee, 2021). This indicates that, at the level of CBUs, specialization is expected to be low.

*Centralization* can be defined as the allocation of decision-making authority within an organization (Mintzberg, 1979). Vertical centralization concerns the distribution of authority across management layers, while horizontal centralization concerns whether authority rests with managers or is delegated to non-managerial professionals. Centralization refers to a structure in which decision-making authority is allocated outside the primary process (e.g. horizontal centralization), often distributed across multiple management layers (e.g. vertical centralization) that are assigned distinct responsibilities, such as resolving issues in the care process, monitoring quality, allocating resources across departments and (re)setting goals. Decentralization indicates that decision-making authority and these management responsibilities are delegated to operational teams. For instance, in healthcare organizations there is often vertical centralization of administrative decisions (e.g. goal-setting, budgeting and resource allocation) across multiple layers of management, alongside horizontal decentralization of clinical decision-making to autonomous professionals, who can also influence administrative decisions through consultation with management (Mintzberg, 1979, 1983). According to Porter and Lee (2021), CBUs ideally hold the authority to initiate and implement improvements in the care process and manage coordination with other units. This reflects decentralization, as decision-making regarding the care process is embedded within the CBU.

### Integrating CBUs within functional structures: opportunities and challenges

The integration of CBUs within the functional structure is different from Porter and Lee's (2021) approach to CBUs, in that they propose a full-system change. From a design theory perspective, the commonly adopted practical approach of integrating a condition-based structure within the functional structure presents specific opportunities and challenges.

From the literature on design theory, we learn that functional structures are characterized by high functional concentration, high specialization and centralization (De Sitter, 1994; Mintzberg, 1979). In these structures, expertise and knowledge are concentrated within specialized departments that provide care for multiple types of conditions or patient groups. Concentrating such expertise in functional departments can help maintain and develop knowledge and facilitate the effective transmission of complex skills (Mintzberg, 1979). Furthermore, centralizing resources and expertise allows scarce and costly resources to be deployed more efficiently, as they can be utilized across multiple processes. In design theory (e.g. Burton *et al*., 2015), the combination of functional departments with cross-functional units, such as CBUs, is known as an approach to enhance the effectiveness of a process, while still leveraging the efficiency of the functional structure. It may therefore be expected that CBUs integrated within a functional structure will benefit from the efficiencies associated with the functional structure characteristics, such as the enhanced development of specialized knowledge within departments and the more efficient deployment of resources.

At the same time, from a design theory perspective, it can be argued that implementing more or less autonomous units, such as CBUs, within a functional structure may be associated with various challenges (e.g. Achterbergh and Vriens, 2019; Christis, 2011b; De Sitter, 1994; Kuipers *et al*., 2018). While such autonomous units are established to manage cross-departmental processes, address their complexity and enhance performance (Kuipers *et al*., 2018), integrating them within a functional structure also requires close alignment with functional departments regarding, for instance, staffing, financial resources and information (Burton *et al*., 2015). This, in turn, increases the need for coordination. Christis (2011b) further argues that distributing responsibilities across both functional departments and these autonomous units may complicate clarity over who is responsible for what. Effective collaboration thus demands intensive coordination between these departments and units to maintain process continuity and quality. From design theory (e.g. Achterbergh and Vriens, 2019; Burton and Obel, 2018; De Sitter, 1994), we learn that such coordination requirements introduce the risk of delays, miscommunication, inefficiencies and other coordination problems.

In summary, based on design theory, we may expect that integrating CBUs within a functional structure can offer opportunities, as the functional structure provides an infrastructure for the efficient sharing of complex knowledge and resources. At the same time, CBUs may face challenges in fostering collaboration and optimizing care processes due to coordination demands. Therefore, this study aims to examine the structure-related opportunities and challenges of CBUs integrated within a functional structure.

## Method

In this study, a qualitative multiple case study design was used (Yin, 2009). This design is particularly suited for our aim, as it enables an in-depth exploration of complex organizational dynamics.

Two Dutch hospitals were purposefully selected using criterion sampling (Patton, 2002). The hospitals were chosen because they (1) had formally integrated CBUs, characterized as multidisciplinary units organized around specific medical conditions to deliver and continuously improve care and (2) had maintained a functional structure, in line with archetypes one and two as described by Wiersema *et al*. (2023). A description of both cases is presented in Table 1 and Table 2.

**Table 1:** Context Hospital A

Hospital A	
Context	Dutch top-clinical hospital 500 beds, 6,300 staff members CBUs incrementally implemented per medical condition or patient group over time, since 2019
Key features CBUs	*Main responsibility*: CBU is responsible for the delivery of care and the improvement of the quality and costs of the care (process) for a specific medical condition or patient group *Leadership*: Led by a medical, nursing and operational leader *Team composition*: All healthcare professionals involved in care for a specific medical condition, including physicians, nurses, allied health professionals, administrative staff; team size varies by condition *Support*: Supported by data analysts, specialists in electronic health record systems and a VBHC advisor
Key features functional hospital structure	The hospital is organized into 13 centers (8 care delivery centers and 3 support centers), each structured around a specific function or medical domain. Centers are led by dual leadership: a medical manager and an operational manager, accountable to the Executive Board Each center consists of multiple functional departments that provide care across various medical conditions. These departments are led by functional managers and are responsible for operations management, productivity and quality policy CBUs are formally embedded within the care delivery centers and report to the dual management of these centers

**Source(s):** Authors’ own work

**Table 2:** Context Hospital B

Hospital B	
Context	Dutch University Medical Center 1065 beds, 12,700 staff members CBUs incrementally implemented per medical condition or patient group over time, since 2022
Key features CBUs	*Main responsibility:* CBU is responsible for the delivery of care and the improvement of the quality and costs of the care (process) for a specific medical condition or patient group *Leadership:* Led either by a medical or a nurse leader alone, or in collaboration with a medical and a nurse leader *Team composition:* All healthcare professionals involved in care for a specific medical condition, including physicians, nurses, allied health professionals and administrative staff; team size varies by condition *Support:* Supported by a decentralized development team, including specialists in electronic health record systems and project support staff
Key features functional hospital structure	The hospital has 41 medical departments, each led by a functional manager and accountable to the Executive Board. These departments focus on care quality and professional development. During the study period, they were also responsible for care delivery and budgeting There are 11 centers organized around medical domains (e.g. oncology, pelvic care, trauma). These centers house the CBUs and are designed to bring together all professionals involved in the care for a specific medical condition (medical specialists, nurses and support staff) to promote integrated, collaborative care. Each center is led by a medical, nursing and operational leader

**Source(s):** Authors’ own work

Following consultation with a manager in Hospital A responsible for the implementation of VBHC and two managers responsible for the development of CBUs in Hospital B, six CBUs were purposively selected using intensity sampling (Patton, 2002). These CBUs were formally embedded within the hospitals' organizational structures and actively involved in delivering and improving care. Both hospitals are gradually implementing units organized around specific medical conditions, introducing new CBUs incrementally. Because the selected CBUs were already implemented within the functional structure, they formed information-rich cases, enabling an in-depth exploration of the opportunities and challenges experienced by CBUs operating within a functional structure. The CBUs are organized around prostate cancer, breast cancer and frail elderly care in Hospital A; and chronic pelvic pain, acute pancreatitis and pulmonary hypertension in Hospital B.

### Ethical considerations and consent to participation

This study was reviewed and approved by the Institutional Review Board of the hospital where two authors (Femke Meijer (FM), and Paul van der Nat (PN)) are employed (Reference No: Z25.023; approved on April 8, 2025) and by the Medical Ethical Commission Utrecht (MEC-U; Reference No: W25.017; approved on February 5, 2025), which concluded that the research does not fall under the Dutch Medical Research Involving Human Subjects Act. All procedures were conducted in accordance with the Netherlands Code of Conduct for Research Integrity (2018). Participants were provided with written information about the aim of the study, assured of the confidential handling of their data and the anonymity of their responses, reminded that participation was voluntary and told that they could withdraw at any time without consequences. Prior to each interview, participants were asked to reconfirm their willingness to participate, and written informed consent was obtained.

### Data collection

Data were collected through semi-structured interviews (*n* = 26) with members of the CBUs. The interview guide used in this study has been included in the supplementary materials. Participants were purposefully selected based on their involvement in the CBUs, targeting the leader(s) of the CBU, healthcare professionals such as nurses and medical specialists and supporting staff. A summary of the participants is provided in Table 3.

**Table 3:** Participants' characteristics

Function	Hospital A (*n* = 12)	Hospital B (*n* = 14)
Medical CBU leader	3	3
Nursing CBU leader	3	1
Operational CBU leader	3	–
Medical specialist	–	4
Nurse (practitioner)	3	4
Support staff	–	2

**Source(s):** Authors’ own work

The first author (FM) conducted the interviews using open-ended questions. Given the abstract nature of the concept of structure, participants were encouraged to speak in a narrative manner about the goals of their CBU, the unit's operational and management tasks in achieving those goals and how these tasks related to those of other departments within the organization. Subsequently, the researcher (FM) shifted the conversation to examining the opportunities and challenges the CBU faced in achieving its goals. For instance, participants were asked to describe how the division of tasks within the care process of the CBU gave rise to opportunities or challenges to optimize patient value and enhance the collaboration between professionals. Furthermore, participants were asked about the CBU's management tasks, including how the CBU initiated and implemented improvements.

### Data analysis

All interviews were audio-recorded and transcribed verbatim. The transcripts were coded by the first author (FM) using ATLAS.ti, following the thematic analysis approach described by Braun and Clarke (2006). A deductive thematic analysis was applied to examine the theory-informed structure dimensions *functional concentration, specialization and centralization* of the CBUs. Specifically, we examined the interviews to understand which operational tasks within the care process were assigned to the CBUs and functional departments, which management tasks they were responsible for and whether these tasks were linked to a single medical condition or multiple types of conditions. Inductive coding was applied to explore how the structure characteristics of the CBUs created opportunities and challenges in achieving their goals. Specifically, we searched for text fragments in which CBU members described how and why the division of tasks, both within the CBU and between the CBU and other hospital departments, resulted in opportunities or challenges in achieving its objectives. Throughout the coding process, three authors (FM, Dorine van Staalduinen (DS) and Dirk Vriens (DV)) continuously discussed and reflected on the interpretation of the codes.

## Findings

In this section, we describe the structure dimensions of the CBUs in hospitals A and B, outline a structure-related opportunity and describe the structure-related challenges resulting from integrating CBUs within the functional structure. If relevant differences between the two hospitals were found, these are explicitly mentioned in the results.

### Structure dimensions for CBUs within a functional hospital structure

Tables 4-6 present the analysis of the structure dimensions of CBUs in hospitals A and B, including functional concentration, specialization and centralization. In both hospitals, CBUs are responsible for the delivery and continuous improvement of the care process for a specific medical condition or patient group. While CBUs are responsible for care delivery, we found that care delivery tasks are specialized across different functional departments. The improvement tasks are carried out within the CBU and cover the entire care process. The CBU initiates improvements for the care process, while functional managers retain responsibility for decision-making, resource allocation and approving CBU-proposed improvements.

**Table 4:** Functional concentration CBUs hospital A and B

	Hospital A	Hospital B
Functional concentration	In both hospitals, the tasks of the CBUs are linked to a clearly defined medical condition (prostate/breast cancer, acute pancreatitis, pulmonary hypertension, chronic pelvic pain patients) or patient group (frail elderly care), indicating *low level of functional concentration at the CBU level*	

**Source(s):** Authors’ own work

**Table 5:** Specialization CBUs hospital A and B

	Hospital A	Hospital B
Specialization	*Provision of care*: Care tasks for conditions addressed by CBUs are mostly divided across multiple functional departments, each responsible for certain steps	
	Some integration occurs, such as combined clinics and task-sharing between departments in the breast and prostate cancer CBU, *reducing specialization in parts of the process*. However, most steps remain *specialized* across departments	In the chronic pelvic pain CBU, intake and diagnosis are integrated into a single process within the CBU, while treatment remains with functional departments. Similarly, the pulmonary hypertension CBU conducts combined diagnostic consultations. This integration *reduces specialization in parts of the care process*. In the acute pancreatitis CBU, most steps in the care process remain *specialized *across departments
	*Improving care process*: The CBUs are responsible for improving care across the entire care process for their medical condition/patient group. Professionals involved in the CBU collaborate to identify and implement improvements that span the entire care process	
	Improvement initiatives are primarily focused on clinical improvements in the care process, and to a lesser extent on organizational improvements to the care process and patient journey	Improvement initiatives are primarily focused on organizational improvements to the care process and patient journey
	These tasks are not limited to a single step or department, which results *in a lower degree of specialization in improvement tasks*	These tasks are not limited to a single step or department, which results *in a lower degree of specialization in improvement tasks*

**Source(s):** Authors’ own work

**Table 6:** Centralization CBUs hospital A and B

	Hospital A	Hospital B
Centralization	In both hospitals, the CBU leaders, together with (healthcare) professionals involved in the CBU, set condition-specific goals, analyze the care process in terms of outcomes and/or costs and initiate improvement efforts	
	Functional managers are responsible for decision-making, budgeting, staffing, and quality control within their departments. The approval and/or implementation of improvements proposed by a CBU that affect a given department rests with these functional managers	
	The distribution of management responsibilities for initiating, approving and implementing improvements across the CBUs and multiple functional managers seems to reflect c*entralization*	

**Source(s):** Authors’ own work

We found that professionals experience benefits from working within a CBU. During the interviews, members of CBUs from both hospitals indicated that working within a CBU improves the coordination of the different steps in care processes and fosters better collaboration among professionals. Specifically, they explained that, because the tasks of the CBU are related to a single medical condition or patient group (e.g. low functional concentration; see Table 4), they were able to develop improvement initiatives tailored to the specific needs and characteristics of that condition.

You can really focus on what matters for this specific condition. You bring everything together and can work toward organizing the entire process as optimally as possible. It's very clear and well-defined, that really helps. - Operational leader, Hospital A

Furthermore, CBU members noted that the broad scope of their improvement task (e.g. improving the care process as a whole) provided them with a more comprehensive understanding of the care process and each professional's role within it. Consequently, they reported improved communication and more efficient information sharing.

We used to work very much within our own silos, right? I did my best for the patient within the scope of surgery, but I was much less aware of what happened earlier in radiology, or what came afterward, for example, in internal oncology. I think that by understanding each other’s work better and actually talking about it, the whole process can become much more efficient. - Medical leader, Hospital A

### Structure-related opportunity: functional departments supporting CBUs

We found that the specialization of certain aspects of care processes across functional departments provides CBUs with opportunities to improve patient value. To reflect on processes and implement improvements, CBUs are supported by medical and non-medical (e.g. information and communication technology) functional departments.

With regard to support from medical departments, a CBU in Hospital A described how a functional department provided the necessary expertise when new professional roles were introduced into the care process. For example, this CBU identified a gap in aftercare and could access a psychiatrist from the organization's functional department when a patient needed support.

A study was conducted, which was really valuable, on how frequently depression occurred within that patient group. We can now use those findings to improve the care we provide to people undergoing hormone therapy, for example by involving psychiatrists from within the hospital. - Nurse, Hospital A

Non-medical functional departments provide CBUs with technical and administrative support. In Hospital A, CBUs collaborate with departments such as data analytics, marketing and communication and VBHC, with each CBU having a dedicated contact person. In Hospital B, CBU members reported having a specialized support team that assists with CBU development, for example, by providing IT support for digital care pathways. These employees not only help CBUs analyze processes and design improvements but also provide insights gained from supporting other CBUs, enabling CBUs to learn from these experiences and enhance their own practices.

CBU members in both hospitals indicated that having a designated point of contact within the functional departments facilitates coordination and collaboration between the CBU and the respective department. They noted that this provides efficient access to the knowledge and expertise required to improve care processes and address individual patient needs, while enabling CBUs to concentrate on their primary care responsibilities rather than supporting tasks.

### Structure-related challenges

#### Ambiguity in decision-making authority in the care process

CBU members explained that it is unclear who holds formal decision-making authority over the care process as a whole. This seems to result from both the specialization of individual steps within the care process across multiple functional departments and the centralization of decision-making authority between the CBU and various functional managers. For instance, improvements that affect the entire care process of a CBU, such as introducing a secretary for the entire CBU or establishing a multidisciplinary patient consultation with two or more specialties present, require coordination and shared resources across departments. While functional managers have decision-making authority over their own process steps, they are not formally responsible for arrangements according to the entire care process of the CBU. Moreover, CBU members expressed the perception that the CBU itself possesses little to no authority to make decisions regarding the care process and lacks control over resources. Although CBUs in Hospital A have established a meeting structure with functional managers, in both hospitals there are no formalized agreements regarding who holds decision-making authority over the care process as a whole. Consequently, the CBU members indicated that CBUs often navigate a fragmented structure in which managers can only make decisions about their own part, while no one holds overarching decision-making authority over the entire care process, as also noted by this nurse leader.

When it comes to improvements, you might discuss something with Manager A, but they can’t make decisions for Group B, so it has to be aligned again with Manager B. But then, who takes the lead? I believe that if one team could work directly with a single contact person, and everyone was aligned, it would be much more effective. - Nurse leader, Hospital A

As a result, decision-making regarding the implementation of improvements at the CBU level seems to be impeded by ambiguity, lack of ownership and limited accountability beyond departmental boundaries. For instance, one CBU sought to modify the sequence of steps within the care process by adding and removing certain activities. The unit's leader explained that it remained unclear who held the mandate to decide on such changes for this particular condition.

In the past, we had three specialties, so patients had to undergo three separate intakes. Now, we want to consolidate them into one intake, which is complicated because it deviates from standard procedures at various outpatient clinics. This raises the question: Who has the authority to decide to work this way for the entire care process of this condition? - Medical Leader, Hospital B

Due to the lack of clarity regarding who holds decision-making authority over changes to the entire care process, it is difficult for CBUs to carry out their responsibility of improving the process, as this medical leader points out.

We want to have a single planner for the entire CBU, but I sense there’s some resistance. We've had managers involved, brought all the operational leaders together, and still we haven’t been able to get it off the ground. - Medical leader, Hospital B

#### Involvement of multiple stakeholders in decision-making processes

We found that the specialization and centralization of decision-making authority lead to many individuals being involved in deciding on and implementing improvements. The CBU members of both hospitals revealed that, since the process steps are embedded within distinct departments, each overseen by a separate manager, CBU improvement efforts targeting the process steps of these departments necessitate the involvement and coordination of multiple managers. Interviewees indicated that the alignment of multiple managers complicates the decision-making process and is a time-consuming process, which can impede the efficiency of the decision-making process and increase the likelihood of delays or non-implementation of improvements. For example, one CBU from Hospital B aimed to introduce preventive actions at various points in the care process, which required the involvement of seven functional managers to adjust existing workflows within their departments. According to the medical leader of this CBU, the need for each manager's approval to modify departmental processes slowed down the implementation of the improvement initiative.

So, if I need additional capacity or feel that an extra medical specialist is needed, I have to go to the managers of that department just to be able to arrange anything. And when multiple specialties are involved, you can easily end up having to deal with five, six, or even seven managers to get something done. - Medical leader, Hospital B

If you do manage to get something done, it can have a real impact. But the groups involved are very large. You’re immediately dealing with many different people when trying to create movement or change something in a process. That makes everything very slow and difficult. - Nurse leader, Hospital A

#### Tension between department goals and CBU goals

The interviews revealed that improvement initiatives proposed by CBUs often appear to be perceived as conflicting with departmental objectives. Due to specialization within the care process, various departments are involved in the different steps of the CBU's care process. CBU members of both hospitals indicated that when their CBU initiates an initiative aimed at improving the entire care process, this may not align with the priorities of the individual departments. As one medical leader from Hospital A explained, a particular diagnostic tool improved the speed and level of detail of diagnostic results, which was highly valuable for treating the condition in question. However, since the investment had to come from the radiology department's budget and the tool did not significantly enhance the department's efficiency, there was less reason to invest. This challenge is further complicated because the departmental procedures are often standardized to serve multiple medical conditions and may not align with the specific needs of one condition.

The CBU members mentioned that the emphasis on departmental responsibilities, rather than the broader care process, can lead to reluctance or resistance, especially in cases where process-wide improvements are seen to compromise the department's performance or resource allocation. For instance, one CBU in Hospital A proposed an initiative to rotate nurses between departments that are linked to the CBU. Functional managers, however, voiced concerns, noting the importance of keeping their staff within their own departments to maintain stability.

The nurses from Department X are sometimes short-staffed, and the nurses from Department Y are willing to help. But then the managers intervene, raising many objections. I think we should first explore what is possible, and only then consider whether it’s truly not a viable option. - Operational leader, Hospital A

We actually want to move [a process step] to the outpatient clinic, but the head of radiology found that difficult. You notice that there are quite a few hurdles to overcome in order to get everyone to think beyond their own department. - Medical leader, Hospital A

## Discussion

This study focused on the organizational impact of VBHC in hospitals by examining the structure-related opportunities and challenges of CBUs integrated within functional structures, corresponding to archetypes one and two described by Wiersema *et al*. (2023). Despite differences in hospital size, bed capacity and staffing, we found that CBUs in the two organizations had similar structure dimensions and structure-related opportunities and challenges. We first reflect on the structure-related opportunity identified in this study and its implications for hospitals' practices. Subsequently, we discuss structure-related challenges and how to address them from a design theory perspective. We then discuss the implications of these findings for VBHC in hospital organizations, as well as for the extant literature on design theory.

### Structure-related opportunity: knowledge transfer and learning across CBUs

Our results show that integrating CBUs within the functional structure enables them to utilize the resources and expertise of both medical and non-medical functional departments. To illustrate, CBUs collaborate with data analysts from the IT department, who contribute the technical skills needed to analyze and improve the care process, or seek support from the psychiatric department to provide additional care for specific patients. These functional departments provide support to multiple CBUs simultaneously. De Sitter (1994) and Christis (2011a) describe this as a form of specialization of support tasks at the strategic level, where support is organized across multiple units, in contrast to the operational level (e.g. support tied to individual tasks) or the structural level (e.g. support tasks at the unit level). In our study, assigning support tasks at the strategic level – having functional departments providing support across multiple CBUs – created opportunities for learning and sharing knowledge between CBUs. Employees in non-medical departments were able to transfer insights, experiences and best practices from one CBU to another by working with multiple CBUs. These findings give context to earlier VBHC research showing how specialized units can foster the transfer of insights and best practices across CBUs (Kaplan *et al*., 2015; Makdisse *et al*., 2018).

Our findings indicate that realizing this opportunity requires effective coordination between the CBU and the specialized departments. Mechanisms from this current study that allow for this coordination are a clear point of contact per specialized department, or a team dedicated to CBUs for project management and process analysis. The importance of such coordination mechanisms is also emphasized in design theory. Burton *et al*. (2015) note that when organizational units share resources, careful alignment across units is nearly as crucial as the resources themselves.

### Structure-related challenges of CBUs in functional structures

Our findings show that CBUs embedded in functional structures face three structure-related challenges in their efforts to integrate and continuously improve the care process. These challenges appear to stem from a lack of formal, integrated authority over the CBU and its processes due to the distribution of care process responsibilities and decision-making authority across multiple functional departments and management layers. Moreover, as we will note in this section, all challenges are related to discussions in organization design theory.

First, the findings reveal that decision-making authority over the CBU's care process is fragmented across multiple functional managers, while the CBU itself has limited authority, leaving no single actor with decision-making authority over the entire care process. This fragmentation creates ambiguity regarding accountability and ownership of the overall care process. In line with organization design research (e.g. Burton *et al*., 2015; Goold and Campbell, 2003; Kuipers *et al*., 2018), the specialization of processes and decision-making is argued to introduce uncertainty about responsibilities and reporting relationships, which may result in the emergence of conflicts or delayed decision-making.

Second, the findings indicate that distributed decision-making throughout the care process requires extensive coordination between CBUs and functional managers. This prolongs improvement trajectories within the CBU. According to design theory scholars (e.g. Goold and Campbell, 2003; Kuipers *et al*., 2018), this stems from the need to build consensus among multiple stakeholders, each of whom has only decision-making authority for a part of the process.

Third, our findings indicate that it is difficult to coordinate decisions with functional managers because departmental processes and routines often conflict with the goals of the overall care process. These tensions are consistent with previous research on organizational structures that combine multiple, potentially conflicting goals (Burton *et al*., 2015; Joseph and Sengul, 2024). As Christis (2011b) argues, this type of structure tends to shift attention away from integrated process outcomes toward the optimization of small, isolated steps. In line with this reasoning, our findings show that functional managers tend to prioritize their own functional step, which constrains cross-departmental collaboration and ultimately limits the CBU to achieve integrated and continuous improvement.

### Addressing structure-related challenges

We draw on design theory to discuss that the challenges may be addressed by two types of mechanisms: integration and a reduction of fragmentation.

### Addressing structure-related challenges through integration

Our findings regarding the challenges indicate that embedding CBUs in a functional organizational structure continues to reproduce fragmentation, thereby impeding effective integration and continuous improvement of care processes. Design theory (cf. Burton *et al*., 2015; Mintzberg and Glouberman, 2001) suggests that in contexts characterized by such fragmentation, additional coordination mechanisms are required to enable integration across separate units and to prevent coordination problems such as those identified in this study. With respect to our findings, two integrative coordination mechanisms seem relevant: clarifying responsibilities and reporting relationships and facilitating cross-departmental professional collaboration.

First, our findings suggest that CBUs introduce responsibilities related to the overall care process that do not align with functionally organized responsibilities and decision-making authority focused on specific parts of the care process. This misalignment gives rise to ambiguity, which in turn necessitates extensive coordination. Design theory literature (cf. Burton *et al*., 2015; Goold and Campbell, 2003) argues that particularly when responsibilities overlap across units – such as functional departments and CBUs – clear mutual agreements are required to clarify roles and set expectations regarding responsibilities. In the context of CBUs, this highlights the need to explicitly redefine responsibilities and assign formal mandates to ensure accountability for decisions affecting the entire care process, thereby preventing ambiguity about who can make decisions or should be involved. And, given that we identified leadership roles established at the CBU level, it seems important to consider the distribution of roles and mandates between former and new CBU management roles as well as the nature of their relationships. Future research could explore management structures for CBU processes, including formal mandates for CBUs.

Second, our findings suggest that CBU goals were not fully aligned with the goals governing departmental work. This created a tension between department-level priorities and CBU-level objectives. In line with Mintzberg (1979, 1983), who argues that hospitals are typical professional bureaucracies in which coordination through output standardization is less effective, solving the tensions between CBU and departmental goals might be realized by fostering a collaborative culture in which functional managers, CBU leaders and care professionals are enabled to reflect on and discuss different goals and to participate in integrated decision-making in which all (CBU and departmental) interests are served (see also Mintzberg (2012), who emphasizes the need for distributed management in healthcare). Rather than relying on traditional vertical control, then, hospital administrators should enable (healthcare) professionals to think and collaborate across departmental and professional boundaries (Mintzberg, 2012). Given that we observed functional managers prioritizing departmental objectives, adopting a facilitative management style in response to CBU requests for care improvement seems critical for managing tensions between departmental and CBU objectives. The lack of formal and integrated authority over the CBU and its processes, as identified in this study, illustrates the risks of introducing coordination structures without corresponding managerial responsibility. Future research could explore how such a management style can be developed and supported in condition-based organizations.

### Addressing structure-related challenges through reducing fragmentation

While some authors in design theory argue for integrative coordination mechanisms to address structure-related challenges, others, such as Christensen *et al*. (2009) and Christis (2011b), argue that such approaches may still fall short of achieving the intended results of organizing care around medical conditions. Coordination mechanisms do not fundamentally resolve the need for complex coordination arising from overlapping tasks or conflicting goals. Instead, these authors identify the functional structure itself as the root cause of coordination challenges and emphasize that true integration of care processes requires a reduction of fragmentation. They argue that a structural intervention toward autonomous units, similar to Porter and Lee's (2021) IPU concept and Wiersema *et al*.’s (2023) archetypes three and four, is necessary to address structure-related challenges. According to Christis (2011b), units that are operationally responsible for a complete process and that are granted decision-making authority facilitate coordination within a single unit. Consequently, the need for complex interdepartmental coordination is reduced, along with associated challenges regarding prolonged decision-making processes, ambiguity and conflicts (Christis, 2011a). When functional departments are retained, Christis (2011b) notes that their role should be limited to collegial consultation without executive or governing responsibilities in the delivery or improvement of care.

Based on this reasoning, our findings indicate that integrating CBUs within functional hospitals requires structural change rather than functioning merely as additional coordination units. From this perspective, CBUs integrated within functional hospitals should, at best, be understood as an intermediate step: granting them greater autonomy and delegating responsibilities could serve as a gradual pathway toward more independent, stand-alone CBUs, similar to IPUs.

### Implications for VBHC literature and design theory

Several important implications for existing literature arise from the findings of this study. First, it contributes to the VBHC literature by providing insights into the practical implications of integrating CBUs within functional hospital structures, an aspect that has received limited attention thus far (Steinmann *et al*., 2022; Wiersema *et al*., 2023). This current research extends the literature by showing that, in order to fully realize the potential of CBUs within a functional structure, hospitals need to be aware of certain challenges that come with this approach and need to take steps beyond merely implementing them within the existing functional structure. These identified challenges show the relevance of insights from design theory, which highlights the importance of additional coordination and integration mechanisms to manage complexity and fragmentation and the need for structural interventions that enable CBUs to operate as independent multidisciplinary teams continuously improving the care process.

Second, this study contributes to design theory (e.g. Achterbergh and Vriens, 2019; Burton and Obel, 2018; De Sitter, 1994) by examining how structure dimensions are operationalized in the healthcare context and the practical boundaries and challenges encountered. Implementing such structures requires balancing practical and institutional feasibility, including financial constraints and cultural aspects, such as maintaining collaboration within long-standing specialist groups, with the aim of VBHC of organizing care around medical conditions. In this context, coordination and collaboration appear to be shaped by both formal structures, with CBUs and condition-based decision-making mandates and professional dynamics, as professionals remain strongly tied to their discipline-specific groups.

## Limitations

A strength of this study is the use of an organizational structure perspective to VBHC, which, to our knowledge, has been rarely applied to examine CBU functioning. This enabled an in-depth and systematic investigation of CBUs in hospitals. Several limitations must be acknowledged. First, the organizational structure perspective leads us to focus on the division of tasks in care delivery and decision-making processes. However, as discussed in the preceding paragraphs, other organizational conditions, such as culture, may also influence the effectiveness of CBUs within a functional hospital structure. Thereby, our results might be limited by their focus on the themes raised in design theory. To further explore organizational aspects that promote or hinder CBUs, other factors such as culture and leadership should be included.

Second, although the study included CBUs from two different hospitals and focused on CBUs from a novel lens, the findings are based on the Dutch healthcare context. This limits the generalizability of the findings. Therefore, future research could explore these and other forms of CBUs in various international contexts to gain a broader understanding of their functioning and development within a functional hospital structure.

## Practical implications

Our findings offer guidance for managers implementing condition-based structures and VBHC. Integrating CBUs within functional hospital structures should be approached as an ongoing learning process, where the functioning and positioning of CBUs are gradually refined. Recognizing that CBUs are not yet fully optimized creates opportunities for organizational learning, which hospital leaders can support by encouraging experimentation with new ways of organizing care around medical conditions.

Based on design theory (cf. Christensen *et al*., 2009; Christis, 2011a, 2011b), managers should ensure that CBUs are operationally responsible for as complete a care process as possible. In the practical context of a functional hospital structure, this involves:

1. *Identifying the functional departments that contribute to a care process and assessing whether these steps can be consolidated within a CBU*. Our empirical findings illustrate the benefits of this approach: in the chronic pelvic pain CBU, integrating diagnosis and treatment planning within a single CBU across multiple specialists enabled simultaneous consultations and direct alignment of decisions and reduced the need for patients and staff to navigate between departments. Granting the CBU leaders decision-making authority for continuous improvement of these care process steps further allows the unit to work more independently and reduces coordination complexity.
2. *Clarifying roles and responsibilities.* When full integration is not feasible due to resources or physical constraints, managers should implement coordination mechanisms, as suggested by Burton *et al*. (2015) and Goold and Campbell (2003), to align distributed processes. In this study, we found that unclear roles and responsibilities often led to delays in decision-making and limited contribution from functional managers to the overall care process. Clarifying the role of functional managers is therefore critical: they should not only manage their own department but also actively support the CBU and the broader patient care process. Explicitly defining these expectations and assigning accountability for cross-departmental outcomes enables functional managers to support improvements that may be locally suboptimal but beneficial overall. In this way, hospitals can better manage tensions between competing goals and accelerate alignment through shared responsibility.
3. *To coordinate relationships between CBUs and functional departments, establish dedicated points of contact within each department for every CBU.* Our study indicates that such points of contact are instrumental in coordinating interdependencies, providing a clear interface for targeted coordination and facilitating alignment and preventing coordination problems, such as ambiguity about who to contact within functional departments.

By systematically integrating the care process within CBUs where possible, implementing coordination mechanisms where necessary and establishing clear roles and points of contact, hospital managers can reduce fragmentation, improve coordination and enable CBUs to achieve their full potential in delivering condition-based care. Future research should examine whether hospital characteristics, such as hospital size or academic versus non-academic status and hospital- or self-employed medical specialists, influence how these changes can be implemented and whether different types of hospitals require distinct approaches to effectively implement the proposed solutions.

## Conclusion

In their transition toward VBHC, many hospitals are integrating CBUs into their existing functional structures. Our study revealed that the structure dimensions of CBUs within a functional structure create both opportunities to leverage the supportive potential of functional departments and challenges related to ambiguity and complexity in responsibilities and decision-making within the care process. The insights gained can inform the development of CBUs integrated within a functional hospital structure. Hospitals can leverage opportunities and mitigate challenges by integrating operational and decision-making responsibilities within CBUs and positioning the functional structure as a supportive infrastructure, thereby reducing process fragmentation and interdependencies between departments and units and lowering the risk of coordination problems. We recommend that hospital managers adopt a learning-oriented, experimental approach to explore and iteratively address the organizational implications of integrating CBUs, thereby gradually developing a condition-based structure.

## Appendix Interview guide

### Introductory question

Can you please introduce yourself and describe your role in the CBU?

### General questions about the CBU

How do you experience working in the CBU?

Could you describe the main goal(s) of the CBU?

To what extent are these goals being achieved?

In your experience, what explains whether or not the goals of the CBU are achieved?

### Theme: Functional concentration

Which patients are served by this CBU?

Are the staff in this CBU dedicated solely to this condition, or do they also carry out tasks for other conditions?

### Theme: Specialization

#### Sub-theme: care process

Can you describe the care process for this patient group?

Who is involved in providing care for this patient group? *Which specialties are involved? Which functional departments are involved within the CBU?*

#### Sub-theme: responsibilities CBU

Which tasks are performed by the CBU for this patient group? *(For example: intake, diagnosis, treatment, aftercare, collecting medical information, administrative tasks* *and* *facility-related tasks)*.

Are there any tasks the CBU is not responsible for?

#### Sub-theme: task distribution

How are tasks distributed among care providers for this patient group?

What do you think of this distribution?

### Theme: Centralization

#### Sub-theme: operational problem-solving

Who holds responsibility for identifying and resolving problems in the patient care process, such as delays, coordination issues, or patient referrals?

#### Sub-theme: improving the care process

How are improvements in the care process designed and implemented within the CBU?

Who is involved in this process?

What is your experience with this improvement process? *Can you give an example of an improvement that was successfully implemented or one that did not achieve the desired effect?*

Can you recall a moment when you noticed that patient care improved because of the CBU?

#### Sub-theme: goal-setting and challenges

How are goals set for the CBU, for example regarding outcomes or costs? *Who is involved?*

What is your experience with this process?

What challenges or problems arise when setting goals, initiating improvements, or resolving issues in the care process?

### Theme: Coordination

#### Sub-theme: dependencies between specialists

How do you experience working together with care providers from other departments/specialties?

Could you share an example of a situation where collaboration went well, or where it proved challenging?

#### Sub-theme: dependencies between departments

In what ways does the CBU collaborate with functional departments? *How is this collaboration organized?*

What is your experience with this collaboration? *Can you give an example of a situation where it worked well or where it was challenging?*

What challenges arise when collaborating with these departments?

Were there specific moments when these problems became apparent? What happened in those situations?

How do functional departments contribute to, or hinder, efforts to improve care within the CBU?

#### Sub-theme: dependencies between CBUs

To what extent do you collaborate with other CBUs, *and in what situations?*

What is your experience with this collaboration?

#### Sub-theme: coordination with formal management

How would you compare the responsibilities of a CBU leader with those of a (functional) manager?

How would you describe the collaboration between the CBU and (functional) managers?

*If the interviewee is a manager or CBU leader: *What is your experience with this collaboration?

### Closing

Thank you for sharing your insights. Do you have any additional thoughts or comments you would like to share?
